# Evaluation of repeated exposure systemic toxicity test of PVC with new plasticizer on rats via dual parenteral routes

**DOI:** 10.1093/rb/rbx020

**Published:** 2018-01-02

**Authors:** Li Hou, Chunguang Fan, Chenghu Liu, Qiujin Qu, Chunren Wang, Yanping Shi

**Affiliations:** 1 Department of Biological Evaluation, Key Lab of Biological Evaluation of Medical Devices, Shandong Provincial Inspection Center for Medical Devices, Jinan 250101, China; and; 2 Department of Biomaterials and Tissue Engineering, National Institutes for Food and Drug Control of China, Beijing 100050, China

**Keywords:** plasticized PVC, repeated exposure systemic toxicity test, dual routes of parenteral administration method

## Abstract

Systemic toxicity caused by repeated exposure to both polar and nonpolar leachables of di(2-ethylhexyl)-1,2-cyclohexane plasticized polyvinyl chloride (PVC) was evaluated with dual routes of parenteral administration method on rats in the study. Experimental group and control group were designed by researchers. Tail intravenous injection with 0.9% sodium chloride injection extracts and intraperitoneal injection with corn oil extracts were conducted to the experimental rats while tail intravenous injection with 0.9% sodium chloride Injection and intraperitoneal injection with corn oil were conducted to the control rats. After 14 days, blood specimens were collected for clinical pathology (hematology and clinical chemistry) analysis. Selected organs were weighed and a histopathological examination was conducted. As a result, compared with the control animals, there were no toxicity-related changes on the parameters above. The results show that the rats do not show obvious systemic toxicity reaction caused by repeated exposure with dual routes of parenteral administration method on rats after administration with both polar and nonpolar exacts of di(2-ethylhexyl)-1,2-cyclohexane plasticized PVC simultaneously up for 14 days.

## Introduction

Polyvinyl chloride (PVC) with good physical properties and good biocompatibility to tissues including blood is widely used in many medical devices such as blood transfusion apparatus, intravascular catheter, blood circuit, heart oxygenator, chest or abdominal drain tube and so on [1]. The plasticizer di-(2-ethylhexyl) phthalate (DEHP) is often used in PVC material, however, the leaching of DEHP to the PVC surface and therefore the risk of entering human body has aroused more and more argument [2–4]. Seeking a safer and more effective plasticizer has become a hot research topic [5]. A new plasticizer, di-(2-ethylhexyl)-1,2-cyclohexane dicarboxylate dicarboxylate (DEHCH) is recently considered as one of the environmental plasticizer to substitute phthalates [6]. According to the ISO10993-11:2009 requirements, the research on repeated contacting body systemic toxicity test was performed on DEHCH plasticized PVC material to verify the safety of using this material continuously [6]. A variety of contacting routes are listed in Appendix A in ISO10993-11:2009 [7], though the most relevant route of administration is often used. For devices with a direct or indirect fluid-path or blood contact environment, extraction using polar extraction vehicle (such as physiological saline) and tests for systemic toxicity by the intravenous route may be appropriate. Although repeated exposure to fat-soluble vehicle may also cause the risk of systemic toxicity, only repeated administration of polar extraction could not fully evaluate the risk of chemical leaching due to the contacting plasma and liquid drug and plasma. Dual routes of parenteral administration method were used in this study to fully evaluate the potential systemic toxicity risk of DEHCH plasticized PVC. In the study, intravenous injection of polar extraction and intraperitoneal injection of nonpolar extraction were observed in the same animals. After duration of 14 days, systemic toxicity response was evaluated according to the experimental results of dual routes administration on the rats.

## Experiments

### Reagents

A dose of 0.9% sodium chloride injection (500 ml, Shandong Hulu Pharmaceutical Co., Ltd., Lot No. 0105062207) is used as polar extract vehicle of DEHCH plasticized PVC and simultaneously as the vehicle control.

Corn oil (Sigma, C8267-2.5 L, Lot No. MKBV2080V) is used as nonpolar extract vehicle of DEHCH plasticized PVC and simultaneously as the vehicle control.

Pentobarbital sodium (Ruitaibio, Lot No. 2015-04-18) is used as anesthetic while humanely anesthetized.

### Instruments and equipment

Automated hematology analyzer (SYSMEX, XT-2000i) is used to test hematology parameters on the whole blood sample obtained from experimental and control animals.

Automatic biochemical analyzer (BECKMAN, AUA680) is used to test clinical chemistry parameters on the serum sample obtained from experimental and control animals.

Automatic blood coagulation analyzer (SYSMEX, CA-7000) is used to test coagulation parameters on the serum sample obtained from experimental and control animals.

Light microscope (OLYMPUS, BX51-32H01) is used for pathology slide observation on organ and tissue obtained from experimental and control animals.

### Test animals

Forty SD rats, 6–9 weeks old, half male and half female, were purchased from Beijing Vital River Laboratory Animal Technology Co., Ltd. The body weight are 182–209 g for female and 224–250 g for male at the beginning of the study.

### Test methods

#### Extraction preparation

In accordance to the requirement in ISO10993-12:2012 [8], the samples were prepared and details are shown in Table 1. The extracts were prepared freshly before administration.

**Table 1:** Extraction preparation methods

Group	Extraction ratio	Extraction temperature	Extraction time	Vehicles/controls
Test	0.2 g/ml	37±1 °C	72±2 h	0.9% sodium chloride injection and corn oil
Control	N/A	37±1 °C	72±2 h	0.9% sodium chloride injection and corn oil

#### Selection and randomization of animals

Animals were selected for the study from a large pool of animals and examined to ensure that they were within the proper age range and body weight range and appeared clinically healthy. The animals were identified and weighed. The animals were randomly assigned to experimental and control groups (10 females and 10 males for each group) and tagged. The initial body weight of both experimental and control groups were checked for statistical significance prior to starting the study. All animals were within ±20% of the mean body weight for both sexes at the start of the study.

#### Administration

The extract of 0.9% sodium chloride injection was given to the experimental animals with 10 ml/kg·BW of volume per dose through continuous intravenous injection to the 10 female rats and the 10 male rats for up to 14 days. The extract of corn oil was given to same experimental animals by intraperitoneal injection with 5 ml/kg·BW of volume per dose every 3 days over the duration of the study. The vehicle solutions were given to the control animals following the same method. The details are shown in Table 2.

**Table 2:** Administration methods

Routes	Dosage volume (ml/kg·BW)	Dose frequency	Rate
Intravenous	10	Daily for 14 days	≤2 ml/min
Intraperitoneal	5	1, 4, 7, 10, 13	Slow bonus

#### Body weights

Body weight records were made to the nearest 1 g weekly during the test.

#### Clinical observations

Clinical observations were conducted daily for general health and mortality.

#### Blood collection

At the day before necropsy, all rats were weighed and fasted for 12–18 h. On the following day, animals were anesthetized by intraperitoneal route with 40 mg/kg of pentobarbital sodium and blood samples were obtained from abdominal aorta. The blood specimens were forwarded to conduct clinical pathology analyses. Parameters and abbreviations are shown in Table 3.

**Table 3:** Parameters and abbreviations of clinical pathology analyses

Hematology	Clinical chemistry analyses
White blood cell (WBC)	Alanine amino transferase (ALT)
Red blood cell (RBC)	Aspartate amino transferase (AST)
Hemoglobin (Hgb)	Alkaline phosphatase (ALP)
Hematocrit (Hct)	γ-glutamyl transpeptidase (γ-GT)
Platelet (PLT)	Total bilirubin (TBIL)
Neutrophils proportion (NEUT%)	Total protein (TP)
Lymphocytes proportion (LYMPH%)	Albumin (ALB)
Monocytes proportion (MONO%)	Glucose (GLU)
Eosinophils proportion (EO%)	Creatinine (CREA)
Basophils proportion (BASO%)	Urea (UREA)
Prothrombin time (PT)	Cholesterol (CHOL)
Activated partial thromboplastin time (APTT)	Triglyceride (TG)
	Calcium ion (Ca^2+^)
	Inorganic Phosphorus (IP)
	Sodion (Na^+^)
	Kalium ion (K^+^)
	Chloridion (Cl^-^)

#### Termination and necropsy

All the rats were euthanized by exsanguinations while anesthetized. A gross necropsy was performed on each animal, which includes examination of the external surface of the body, all orifices and the cranial, thoracic and abdominal cavities and their contents. The testes/ovaries, uterus/epididymises, spleen, liver, adrenals, kidneys, thymus, heart and brain was weighed wet as soon as possible after dissection. The following organs were preserved in 10% formalin until further processing: testes/ovaries, uterus/epididymises, spleen, stomach, intestine, pancreas, liver, adrenals, kidneys, thymus, heart, lung, thyroid gland and brain.

#### Histopathology

The fixed tissues were embedded, cut and stained for pathology analyze.

#### Evaluation and statistics

Body weight data, hematological and clinical chemistry data and organ/body weight ratios were evaluated statistically by comparing the experimental group with the control group (SPSS17.0). Data from males and females were analyzed separately. Calculations resulting in probability (*P*) values <0.05 were considered as statistically significant. The findings in the study were comprehensive analyzed according to the results of all the test items.

## Results

### Mortality

During the test, no animal was died in the experimental or the control group.

### Clinical observation

All rats appeared healthy and clinically normal over the course of the study. There were no behavioral changes related to the toxicity of the test article in the experimental groups.

### Body weight

**Figure 1:**
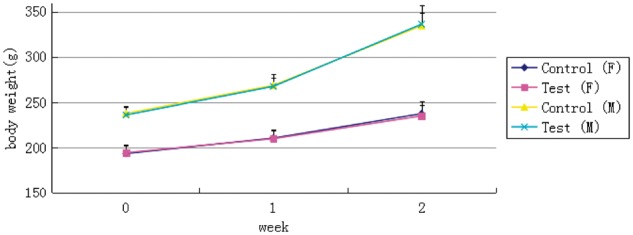
Weight trend line chart

There were no significant differences in body weights between the experimental and control groups of two sexes during the study(*P*>0.05). Data are presented in Fig. 1 and Table 4. As can be seen from Fig. 1, the weight of rats in different sexes showed a trend of increase, which was consistent with that of the control group.

**Table 4:** Summary of body weight data (g, mean±SD)

Week	Control (F)	Test (F)	Control (M)	Test (M)
Week 0	193.7±9.7	194.7±7.9	238.0±7.3	236.2±9.4
Week 1	210.8±8.7	210.1±10.3	268.9±8.1	267.7±13.2
Week 2	237.7±13.4	235.2±11.5	334.5±14.6	336.2±20.4

F: Female, M: Male.

### Clinical pathology

The hematology and clinical chemistry values showed no evidence of systemic toxicity and were similar between the experimental and control groups with a few exceptions. The values for those parameters were within a normal range and the differences were not considered biologically meaningful. Data are presented in Tables 5 and 6.

**Table 5:** Summary of hematology data (mean±SD)

Parameters	Control (F)	Test (F)	Control (M)	Test (M)
WBC (10^9^/l)	3.73±1.25	4.30±0.95	4.44±1.61	4.35±1.23
RBC (10^12^/l)	6.57±0.34	6.45±0.21	6.93±0.30	6.68±0.37
Hgb (g/l)	127.20±4.78	125.50±3.41	134.70±4.32	129.30±7.21
Hct (%)	36.98±1.45	36.36±0.90	39.42±1.12	38.05±1.93
PLT (10^9^/l)	1074.70±83.33	1040.20±51.54	1140.50±108.79	1163.20±129.66
NEUT (%)	14.11±6.77	7.91±4.37^a^	7.83±3.20	6.64±2.06
LYMPH (%)	75.88±7.11	81.95±4.45^a^	82.10±3.36	82.69±2.68
MONO (%)	8.75±1.94	9.11±2.38	9.46±1.22	10.19±2.20
EO (%)	1.26±0.69	1.03±0.63	0.59±0.12	0.48±0.28
BASO (%)	0.00±0.00	0.00±0.00	0.02±0.06	0.00±0.00
PT (s)	8.16±0.31	8.11±0.27	8.94±0.61	9.06±0.30
APTT (s)	21.39±4.63	20.57±5.00	21.03±3.91	21.50±4.40

a *P*<0.05.

**Table 6:** Summary of clinical chemistry data (mean±SD)

Parameters	Control (F)	Test (F)	Control (M)	Test (M)
ALT (U/l)	51.67±49.14	35.93±12.15	44.68±11.83	44.69±7.93
AST (U/l)	150.66±89.39	132.74±30.99	137.68±24.19	142.30±25.18
ALP (U/l)	103.88±21.16	90.98±19.58	232.80±37.72	233.83±36.30
γ-GT (U/l)	0.53±0.42	0.49±0.74	0.72±0.31	0.97±0.27
TBIL (µmol/l)	0.48±0.23	0.48±0.21	0.39±0.15	0.32±0.19
TP (g/l)	57.00±3.58	57.12±5.20	51.46±2.18	52.09±1.91
ALB (g/l)	34.61±2.21	34.53±3.33	29.45±1.22	29.17±1.18
GLU (mmol/l)	6.59±0.34	7.08±0.50^a^	6.43±0.61	6.58±0.47
CREA (µmol/l)	25.68±3.09	27.71±2.12	22.80±2.24	22.83±1.16
UREA (mmol/l)	5.29±0.67	5.98±0.95	4.24±0.45	4.37±0.42
CHOL (mmol/l)	1.32±0.22	1.49±0.38	1.50±0.30	1.53±0.25
TG (mmol/l)	0.26±0.05	0.31±0.09	0.36±0.15	0.29±0.10
Ca^2+^ (mmol/l)	2.26±0.06	2.24±0.08	2.19±0.05	2.18±0.05
IP (mmol/l)	2.46±0.20	2.36±0.07	2.77±0.13	2.80±0.17
Na^+^ (mmol/l)	139.58±1.75	140.13±1.05	142.20±0.83	141.68±0.71
K^+^ (mmol/l)	4.09±0.24	4.04±0.22	4.33±0.18	4.28±0.22
Cl^-^ (mmol/l)	104.02±1.46	104.18±1.00	104.71±1.34	103.61±1.40

a *P*<0.05.

### Organ/body weight ratios

Organ/body weight ratios were similar between the experimental and control groups with a few exceptions. But relevant clinical observations, clinical pathology, necropsy and histopathology examination did not reveal any signs of abnormality or toxicity. Therefore, the differences were not considered biologically meaningful. Data are presented in Table 7.

**Table 7:** Summary of organ/body weight ratios data (mean±SD)

Organs	Control (F)	Test (F)	Control (M)	Test (M)
Brain	0.750±0.065	0.761±0.035	0.585±0.042	0.573±0.036
Thymus	0.168±0.017	0.182±0.032	0.182±0.035	0.161±0.028
Heart	0.308±0.021	0.322±0.026	0.334±0.036	0.306±0.025
Liver	2.834±0.178	2.873±0.239	2.713±0.150	2.833±0.106^a^
Spleen	0.220±0.028	0.269±0.037^a^	0.202±0.031	0.222±0.034
Adrenal	0.027±0.002	0.027±0.004	0.015±0.003	0.013±0.002
Kidneys	0.772±0.058	0.757±0.035	0.734±0.037	0.766±0.033
Ovaries/testes	0.053±0.009	0.053±0.011	0.908±0.075	0.873±0.072
Uterus/epididymises	0.237±0.091	0.250±0.078	0.228±0.046	0.193±0.013

a *P*<0.05.

### Necropsy

**Figure 2:**
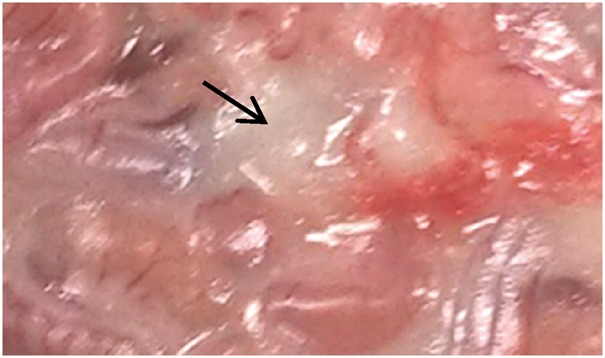
The oil retained in the abdominal cavity at necropsy after 14 days of administration, as shown by the arrow

Small amounts of unabsorbed ivory-white oils could be seen in the abdominal cavity in experimental group and control group animals (Fig. 2), which means that every 3 days of intraperitoneal injection of corn oil cannot make it completely absorbed over the duration of the study. There were no macroscopic changes at necropsy in the two groups.

### Histopathology

Observed under a microscope, the liver cells arranged in a neat way, liver cell morphology is normal, no hepatocellular degeneration and necrosis, there was no fibrous knot tissue hyperplasia in mesenchyme and no inflammatory cell infiltration and liver blood Sinus dilation. The above pathology results mean that there were no relevant histopathology findings of the treatment on histopathology evaluation in liver tissue.

Glomerular morphology is normal and there were no epithelial cell degeneration, necrosis and no fibrous connective tissue hyperplasia or inflammatory cell infiltration. Glomerular capillary endothelial cells and mesangial cells showed no swelling or hyperplasia. The above pathology results mean that there were no relevant histopathology findings of the treatment on histopathology evaluation in kidney tissue.

There were no degeneration, necrosis or shedding of the epithelial cells of the seminiferous tubules in testicular tissue, and the spermatogenic cells were arranged in different stages of development. No interstitial infiltration and inflammatory cell infiltration were found. The above pathology results mean that there were no relevant histopathology findings of the treatment on histopathology evaluation in testicle tissue.

**Figure 3:**
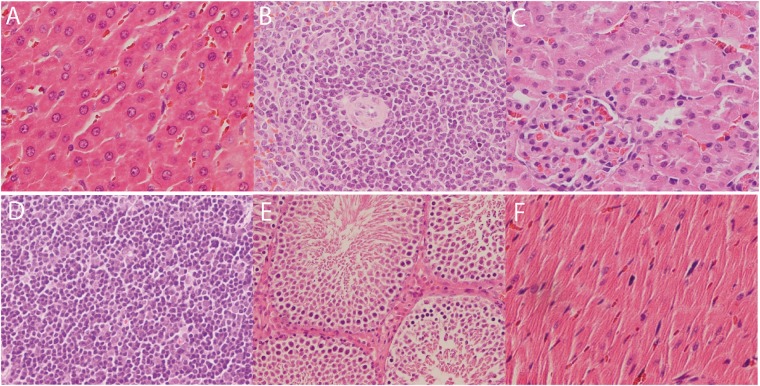
The organ histopathology pictures of the treatment group. (**A**) Liver (HE, ×200), (**B**) spleen (HE, ×400), (**C**) kidney (HE, ×400), (**D**) thymus (HE, ×400), (**E**) testicle (HE, ×200), (**F**) heart (HE, ×400)

There were also no relevant histopathology findings of the treatment on histopathology evaluation in spleen, thymus, heart. Pathological results show that DEHCH does not produce liver toxicity, nephrotoxicity, testicular toxicity and other organ toxicity in this study. The organ histopathology pictures of liver, spleen, kidney, thymus, testicle, heart are shown in Fig. 3.

## Discussion

The toxic and carcinogenic effects of DEHP have been well established in experimental animals about the ability of this compound to produce adverse effects especially liver toxicity, testicular toxicity [9, 10]. As a new type of plasticizer, the focus of di(2-ethylhexyl)-1,2-cyclohexane DEHCH is not only on the short-term compatibility including acute toxicity but on the potential impact of repeated contact toxicity, reproductive toxicity, genetic toxicity [11]. In the experimental design, full consideration should be given to influence factors of toxicity such as the clinical use of material factors, clinical contact and contact time. PVC material is mostly used for external communicating devices which serve as a conduit for entry into the vascular system or contact circulating blood, so extraction preparation method is particularly important before performing biocompatibility test, especially the use of polar and nonpolar solvent vehicles. Both vehicles help to evaluate the toxicity of full extraction of the water-soluble and fat-soluble components into the human body effect on target organs generated.

According to the characteristics of PVC materials plasticized by DEHCH, we pioneered the use of polar and nonpolar vehicles and injected into test rats in the dual administration routes, which simulates the effects of clinically applicable water-soluble and fat-soluble drugs on PVC plasticized by DEHCH. This test method has an advantage over the traditional long-term repeated exposure toxicity and will be more comprehensive and objective evaluation of DEHCH plasticized PVC materials. The corn oil will have a certain impact on the body when long-term continuous exposure [12], but no experimental liver toxicity, nephrotoxicity, testicular toxicity in male rats and other organ toxicity in tissue pathology and clinical pathology influence on the rats when exposure the body on the interval of 3 days by intraperitoneal injection of corn oil excepting that intraperitoneal unabsorbed oily medium was found in test and control groups. The polar and nonpolar media in the protocol do not have interference effect on the test system of repeated exposure systemic toxicity. However, further research is needed to verify whether exposure frequency and dosage of nonpolar extract on rats besides the dual parenteral routes design on the same animals has been maximized to reflect the actual hazard of clinical use.

## Conclusion

Systemic toxicity caused by repeated exposure to both polar and nonpolar leachables of di(2-ethylhexyl)-1,2-cyclohexane plasticized PVC was evaluated with dual routes of parenteral administration method on rats. In this study, DEHCH plasticized PVC materials did not appear to cause a specific change in toxicity. Based on the obtained data, a new test method for the potential toxic effects of DEHCH may be set up, after the future clinical safety is confirmed.
